# The Role of Diverse Liver Cells in Liver Transplantation Tolerance

**DOI:** 10.3389/fimmu.2020.01203

**Published:** 2020-06-12

**Authors:** Yanzhi Jiang, Weitao Que, Ping Zhu, Xiao-Kang Li

**Affiliations:** ^1^Division of Transplantation Immunology, National Research Institute for Child Health and Development, Tokyo, Japan; ^2^Guangdong Cardiovascular Institute, Guangdong Provincial People's Hospital, Guangdong Academy of Medical Sciences, Guangzhou, China

**Keywords:** allograft, hepatic microenvironment, liver transplantation, T cell, tolerance

## Abstract

Liver transplantation is the ideal treatment approach for a variety of end-stage liver diseases. However, life-long, systemic immunosuppressive treatment after transplantation is required to prevent rejection and graft loss, which is associated with severe side effects, although liver allograft is considered more tolerogenic. Therefore, understanding the mechanism underlying the unique immunologically privileged liver organ is valuable for transplantation management and autoimmune disease treatment. The unique hepatic acinus anatomy and a complex cellular network constitute the immunosuppressive hepatic microenvironment, which are responsible for the tolerogenic properties of the liver. The hepatic microenvironment contains a variety of hepatic-resident immobile non-professional antigen-presenting cells, including hepatocytes, liver sinusoidal endothelial cells, Kupffer cells, and hepatic stellate cells, that are insufficient to optimally prime T cells locally and lead to the removal of alloreactive T cells due to the low expression of major histocompatibility complex (MHC) molecules, costimulatory molecules and proinflammatory cytokines but a rather high expression of coinhibitory molecules and anti-inflammatory cytokines. Hepatic dendritic cells (DCs) are generally immature and less immunogenic than splenic DCs and are also ineffective in priming naïve allogeneic T cells via the direct recognition pathway in recipient secondary lymphoid organs. Although natural killer cells and natural killer T cells are reportedly associated with liver tolerance, their roles in liver transplantation are multifaceted and need to be further clarified. Under these circumstances, T cells are prone to clonal deletion, clonal anergy and exhaustion, eventually leading to tolerance. Other proposed liver tolerance mechanisms, such as soluble donor MHC class I molecules, passenger leukocytes theory and a high-load antigen effect, have also been addressed. We herein comprehensively review the current evidence implicating the tolerogenic properties of diverse liver cells in liver transplantation tolerance.

## Introduction

Liver transplantation is the ideal therapeutic approach for a variety of end-stage liver diseases. However, life-long, systemic immunosuppressive treatment is required after transplantation to prevent rejection and graft loss, which is associated with high costs and severe side effects, including infections and malignancy (1, 2). From an immunological standpoint, a liver allograft is more tolerogenic than such grafts for other solid organs, like the heart, kidney, and lung. Spontaneous liver allograft acceptance without the need for immunosuppression has been observed in multiple experimental animal transplantation models (3–5). In clinical practice, liver allografts show a lower rejection rate than such grafts of other solid organs, and around half of carefully selected liver transplant recipients are able to be completely weaned from immunosuppression, which rarely occurs in cases of other organ transplantation (5–7). Furthermore, liver allografts are associated with tolerance induction for other simultaneous or sequentially transplanted organs in human and animal models, indicating that the liver can induce systemic tolerance (8–12). Therefore, understanding the mechanisms underlying the unique immunologically privileged liver organs is valuable for transplantation management and autoimmune disease treatment.

The liver is the central metabolic organ responsible for metabolism, nutrient storage and detoxification and also functions as an immunological organ. To fulfill its multifaceted functions, the liver comprises repetitive functional units formed by a myriad of cell types. The functional unit, known as the hepatic acinus, consists of an irregular-shaped, roughly ellipsoidal mass of parenchymal cells grouped around the terminal branches of hepatic arterioles and portal venules just as they anastomose into sinusoids (13, 14). The liver sinusoids are lined by a thin layer of fenestrated liver sinusoidal endothelial cells (LSECs) and lack organized basal lamina, which facilitate the passage of blood plasma to the underlying hepatocytes. Microvilli of hepatocytes extend into the space of Disse, existing between sinusoids and hepatocytes and exerting metabolic functions.

The liver receives a dual blood supply from the hepatic artery and portal vein. The arterial blood is oxygenated, while the venous blood is rich in pathogens, toxins and harmless dietary antigens from the gut; the liver therefore faces constant immunologic challenges. The arterial and portal-venous blood undergoes confluence and runs through the liver sinusoids toward the central vein or terminal hepatic venules at a low speed, which facilitates the uptake of gut-derived content by liver cells. As an important barrier between the gut and the circulation, the liver interstitium is highly enriched in both innate and adaptive immune cells, such as LSECs, Kupffer cells (KCs), dendritic cells (DCs), hepatic stellate cells (HSCs), natural killer (NK) cells, natural killer T (NKT) cells, and T cells. These cells contribute to the formation of a local tolerogenic milieu that ignores most harmless self and foreign antigens while retaining immunity to pathogens in order to maintain immune system homeostasis. The overall tolerogenic properties of the liver are markedly manifested in the era of transplantation.

**Figure 1:**
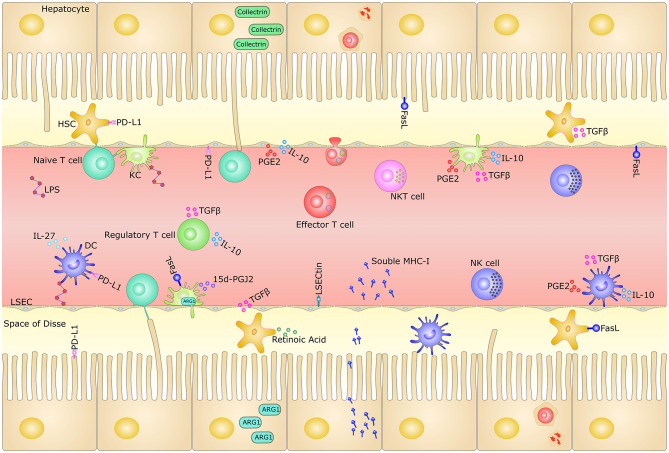
Mechanisms of tolerogenic hepatic microenvironment. The liver sinusoids are lined by a thin layer of fenestrated LSECs and lack organized basal lamina, which facilitate the passage of blood plasma to the underlying hepatocytes. Microvilli of hepatocytes extend into the space of Disse, existing between sinusoids and hepatocytes. The oxygenated arterial and nutrient- rich portal-venous blood undergoes confluence and runs through the liver sinusoids, carrying pathogens, toxins and harmless dietary antigens from the gut. The liver is highly enriched in both innate and adaptive immune cells, such as LSECs, KCs, DCs, HSCs, NK cells, NKT cells, and T cells. The unique liver microenvironment, with its slow blood flow and fenestrated endothelium in the narrow hepatic sinusoids, permits the continuous functional interaction between circulating naive T cells and the diverse hepatic-resident immobile non-professional APCs, such as hepatocytes, LSECs, KCs, DCs, and HSCs. This makes the liver the only non-lymphoid organ that can prime naïve T cell locally. These cells contribute to the liver tolerance through direct cell-cell interaction signaling by surface inhibitory molecules, as well as immunosuppressive milieu through secretory factors. The hepatocytes could also release massive amounts of soluble MHC class I molecules and destroy activated CD8^+^ T cells through “suicidal emperipolesis” mechanism. HSC, hepatic stellate cell; LSEC, liver sinusoidal endothelial cell; DC, dendritic cell; NK, natural killer cell; NKT, natural killer T cell; KC, Kupffer cell; IL-10, interleukin (IL)-10; TGF-β, transforming growth factor (TGF)-β; LSECtin, LSEC C-type lectin; MHC-I, major histocompatibility complex class I; PD-L1, programmed death ligand 1; FasL, Fas ligand; LPS, lipopolysaccharide; IL-27, interleukin (IL)-27; 15d-PGJ2, 15-Deoxy-Delta-12,14-prostaglandin J2; ARG1, Arginase-1; APC, Antigen-presenting cell; PGE2, Prostaglandin E2.

We herein comprehensively review the current evidence implicating the tolerogenic properties of diverse liver cells in liver transplantation tolerance (Figure 1).

## Hepatic Parenchyma Mediated Tolerance Effects

### Role of Hepatocytes

Approximately 60–80% of the total liver cell population is composed of parenchymal hepatocytes, which robustly express and secrete large amounts of proteins involved in metabolism, glycogen synthesis and toxin decomposition (15, 16). There is growing evidence showing that hepatocytes are involved in immunity by expressing immune receptors, such as pattern recognition receptors, major histocompatibility complex (MHC) and adhesion molecules (16–18). The special physiological and immunological functions of hepatocytes and their complex interaction with non-substantive cells of the liver have a significant impact on the host's immune system and can promote immune tolerance in cases of liver transplantation.

The microvilli of hepatocytes can make contact with the filamentous pseudopodia of T cells across the endothelial fenestrations, thereby presenting antigens to T cells (19, 20). Hepatocytes continuously express MHC class I and are capable of presenting antigens to CD8^+^ T cells to trigger CD8^+^ T cell activation and proliferation (21). Hepatocytes can alternatively present antigens to CD8^+^ T cells through cross-presentation, which is controlled by a specific molecular chaperone called collectrin in the endoplasmic reticulum-Golgi intermediate chamber (22). However, due to the lack of necessary survival factors, CD8^+^ T cells activated by hepatocytes quickly undergo apoptosis through BCL-2-interacting mediator (bim)- and caspase-dependent apoptosis after transient proliferation and cytotoxic T lymphocyte (CTL) function (20, 23). Hepatocytes can also actively induce CD8^+^ T cell apoptosis via the FAS or TNF pathway (24). Furthermore, hepatocytes primed CD8^+^ T cells produce abundant amounts of interleukin (IL)-10 in the absence of IFN-β-producing NKT cells co-activated by the same hepatocytes, thus exerting immunosuppressive function (25). When confronted with an inflammatory response, hepatocytes can be induced to express MHC class II and present antigens to CD4^+^ T cells (26, 27). Hepatocytes were found to mediate the Th2 differentiation of uncommitted CD4^+^ T cells and abrogate the capacity of established Th1 cells to secrete IFN-γ (28). Interestingly, hepatocytes promote the conversion of CD4^+^ T cells into CD4^+^CD25^+^Foxp3^+^ regulatory T (Treg) cells and thus induce immune tolerance through the Notch signal pathway (29). Moreover, exosomes or paracrine factors secreted by hepatocytes can also be involved in immune tolerance by interacting with lymphocytes (30, 31).

In brief, hepatocytes regulate immune tolerance in liver transplantation directly and indirectly, and more studies in the future are needed to clarify the mechanism underlying hepatocyte-mediated immune tolerance.

## The Innate Immune Tolerance Mechanisms

### Role of LSECs

LSECs constitute about 50% of non-parenchymal cells in the liver and line the hepatic sinusoids (16). Due to the special structure and abundant blood supply of hepatic sinusoids, LSECs filter out antigens in the blood and play a vital role in maintaining the homeostasis of the hepatic immune microenvironment (32). LSECs express a variety of recognition receptors and scavenger receptors to clear away pathogens in a non-specific manner thus to maintain immune homeostasis of the liver (33–36). In addition, LSECs express MHC class I and II to present antigens to CD8^+^ and CD4^+^ T cells, acting as important hepatic resident non-professional APCs (32, 34, 37, 38). On the other hand, LSECs collect MHC class I molecules from their neighbor cells for cross-presentation to CD8^+^ T cell (39).

LESCs primed naïve CD4^+^ T cells toward Treg differentiation and suppressed the Th1 and Th17 function via IL-10 and PD-1 signaling (33, 38, 40). Studies have shown that LSECs promote the growth of IL-4-expressing Th2 cells and induce a mass of IL-10 secretion through the Notch pathway, thereby creating an immunosuppressive environment within the liver (41). Furthermore, LSECs are able to induce CD4^+^ T cells apoptosis via the Fas/FasL pathway (42). LSECs-mediated CD8^+^ T cell tolerance is antigen-dose-dependent, meaning that low-dose cross-presenting antigens induces immune tolerance, while high-dose induces effector T cells (43). CD8^+^ T cells activated by LSECs may exhibit a distinctive phenotype of CD25^low^CD44^high^ CD62L^high^, which fails to show specific cytotoxicity *in vivo* (44). The interaction of LSECs with naïve CD8^+^ T cells would in turn promote the tolerogenic maturation of LSECs, characterized by increased expression of MHC class I and programmed death ligand 1 (PD-L1). LSECs can also induced CD8^+^ T cells apoptosis in a PD-L1 -dependent manner (44). Besides, researchers found that LSEC C-type lectin secreted by LSECs negatively regulates the immune response by specifically recognizing activated T cells via CD44 (45, 46).

### Role of KCs

KCs are liver-resident macrophages and account for one-third of the non-parenchymal cells in the liver and almost 90% of all residential macrophages in the body (47). Under physiological conditions, KCs are maintained by self-renewal from local precursors, whereas in response to inflammation, KCs are differentiated from infiltrated bone marrow-derived monocytes. KCs predominantly reside in the periportal region of the sinusoidal lumen, where they are optimally located to respond to systemic or gut-derived antigens and circulating immune cell populations. KCs are equipped with an array of scavenger receptors, Toll-like receptors, complement receptors and Fc receptors through which they detect, bind and internalize pathogens, accompanied by the production of cytokines and chemokines, such as tumor necrosis factor-α (TNF-α), IL-1β, IL-6, IL-12, and IL-18 (37, 48, 49). Under steady-state conditions, KCs also serve as tolerogenic APCs by expressing low levels of MHC class II molecules and costimulatory molecules and secrete anti-inflammatory mediators, such as IL-10, transforming growth factor (TGF)-β1, nitric oxide, or prostaglandin E2, which can suppress antigen-specific T cells activation (50–53). KCs also strongly express the coinhibitory molecules programmed death (PD-1) and PD-L1, which can also inhibit the proliferation and functions of T cells by directly contacting them (54, 55). Furthermore, the interplay between KCs and hepatic Tregs is critical for IL-10 production and the induction of systemic T cell tolerance to hepatocyte-derived antigens (56). The role of KCs in organ transplantation induction has long been implicated in animal transplantation model (57–59). Early studies reported that KCs could contribute to absorption and subsequent clearance of alloreactive antibodies (60, 61). More recently, Chen et al. demonstrated that the deletion of graft KCs using gadolinium trichloride prevented the apoptosis of recipient T cells and consequently spontaneous graft acceptance in a rat liver transplantation model. The apoptosis of T cells induced by KCs was related to nuclear factor kappa B (NF-κB) activity and the Fas/FasL pathway, which was associated with spontaneous liver tolerance (62). However, when this approach was examined in a mouse liver transplantation model, the deletion of graft KCs using clodronate liposomes retained liver allograft acceptance (63). It is also worth to note that in the setting of transplantation, a large proportion of donor-derived KCs are being substituted by recipient-derived macrophages over time after transplantation. The recipient-derived macrophages are thought to be more immunogenic and thus able to promote graft pathology (55, 64, 65).

### Role of Liver DCs

DCs are professional APCs that play critical roles in the instigation and regulation of immune responses (66, 67). The general ontogeny, function and classification have been well-described elsewhere (68, 69). The liver harbors more interstitial DCs than any other non-lymphoid organs, including classical myeloid DCs (mDCs) and plasmacytoid DCs (pDCs) (70). They predominantly reside around the portal triad and central vein, with a few cells scattered interstitially between hepatocytes. Due to continuous *in situ* exposure to gut-derived factors, freshly isolated murine hepatic DCs are resistant to lipopolysaccharide (LPS)-mediated maturation, which is termed the endotoxin tolerance phenomenon and is also observed in macrophages/monocytes (71, 72). Compared with secondary lymphoid tissue DCs, freshly isolated hepatic DCs are immature and less immunogenic, express low levels of MHC class II and costimulatory molecules (CD80 and CD86) and secrete low levels of IL-12 (73–76). They prefer to produce IL-10 and IL-27 in response to LPS (77) and are less effective in priming naïve allogeneic T cells and Th1 skewing while favoring Th2 cell polarization (71, 73, 78, 79). Human hepatic DCs favor the generation of Th2 cells and Tregs through an IL-10-dependent mechanism (80, 81). The liver is particularly enriched in pDCs, which can suppress effector T cells through IL-27/Stat3 pathway-dependent PD-L1 expression and induce IL-10-producing Tregs via inducible costimulatory ligand (ICOS-L) expression (82, 83).

DCs were thought to be key mediators in spontaneous hepatic allograft tolerance due to their central roles in regulating the immune response. The trigger of allograft immunity relies on three antigen recognition pathways: the direct pathway, indirect pathway, and semi-direct pathway (84, 85). Donor hepatic DCs quickly migrate to the recipient graft-draining lymphoid tissues as passenger leukocytes, where they directly present intact, donor (allogeneic) MHC molecules to alloreactive T cells. The direct allorecognition pathway is considered the dominant pathway of acute rejection. Although this phenomenon exists in almost all types of organ transplantation, the phenotype and function of donor DCs determines the fate of alloreactive T cells, resulting in either graft tolerance or graft rejection. The tolerogenic properties of hepatic DCs may tilt the balance toward graft tolerance. Liver allografts were acutely rejected when donor hepatic DCs were depleted using a CD11c-DTR mouse model before transplantation (86). However, when the interstitial DC quantity was significantly increased by FMS-like tyrosine kinase 3 ligand (Flt3L) treatment of the donor, liver allografts were also rejected acutely (87, 88). Acute rejection is associated with a marked IL-12 reduction by donor DCs. IL-12 neutralization enhanced the apoptotic death of T cells within both the grafts and the spleen and prolonged the survival of grafts from Flt3L-treated donors. Donor grafts from DAP12^−/−^ mice, whose mDCs exhibit a more mature phenotype than that of naïve mice with enhanced migratory and T cell allostimulatory abilities, failed to induce tolerance and were rejected acutely (89).

Following transplantation, donor-derived hepatic DCs were quickly diminished and replaced by recipient DCs, which peaked on post-operative day 7 and persisted indefinitely. These recipient DCs acquired and expressed intact donor MHC molecules via cell-cell contact or extracellular vesicles and were thus termed cross-dressed DCs (90–94). Interestingly, around 60% of host DCs in liver grafts are cross-dressed DCs. They express high levels of PD-L1 and IL-10, subvert the host anti-donor T cell responses and promote liver transplantation tolerance (95). In contrast, the non-cross-dressed DCs show a minimal suppressor function.

Although the role of DCs in spontaneous hepatic allograft tolerance remains to be further investigated, the manipulation of DCs, such as by *in situ* targeting or infusion after *ex vivo* generation, has been shown to be a promising approach for promoting donor-specific tolerance. The *ex vivo* generation of regulatory DCs can be achieved by culturing DC progenitors using low concentrations of granulocyte-macrophage colony-stimulating factor (GM-CSF) ± IL-4, with the addition of one or more pharmacological agents, such as IL-10, dexamethasone, Vitamin D3, or rapamycin (96–98). The *in situ* manipulation of DCs, such as by the delivery of immunomodulatory factors targeting DCs to regulate alloreactive T cell responses, is an alternative approach to achieve donor-specific transplantation tolerance (99). In experimental transplantation models, the manipulation of DCs showed encouraging efficacy and safety in organ-specific tolerance induction (99–101). Several early-phase clinical trials of *ex vivo*-generated DCs in living-donor liver transplantation have recently been initiated (clinicaltrials.gov identifier: NCT03164265 and NCT04208919) (99, 102).

### Role of HSCs

HSCs account for about 5–8% of liver non-parenchymal cells (103). HSCs are distributed in the space of Disse, in which the cytoplasm is rich in retinoid lipid droplets and vitamin A and regulate the blood flow in the sinusoids of the liver. HSCs undergo activation in response to liver injury and inflammatory events (104, 105). Activated HSCs secrete cytokines, chemokines and extracellular matrix to participate in the pathogenesis of liver fibrogenesis.

The HSCs are potent liver-resident APCs that have the ability of tolerizing T cells. They can induce T cells apoptosis through the PD-L1, B7-H4, and the Fas/FasL signaling pathways and veto the activation of CD8^+^ T cells through a CD54-dependent pathway, thereby suppressing the T cell immune response and maintaining homeostasis and tolerance in the liver (106–111). In a mouse islet transplantation model, co-transplantation of HSCs and islet cells reduced the rejection rate and prolonged the survival of the graft through TRAIL-mediated T cell apoptosis and reduced immune cell infiltration in the graft (112, 113). Activated HSCs induce the conversion of mature monocytes into myeloid-derived suppressor cells (MDSCs), which may contribute to liver immunosuppression (114, 115). In addition, HSCs also participate in immune tolerance by secreting the immunosuppressive factors TGF-β1 and *all-trans* retinoic acid, thereby promoting the differentiation and proliferation of Foxp3^+^ Tregs (116–119). In liver transplantation models, activated HSCs induced immune tolerance by inducing T cell apoptosis and stimulating IL-10 and TGF-β1 production (110). Activated HSCs also promote transplantation tolerance by inducing selective expansion of allogeneic Tregs and reducing inflammation and alloimmunity (117).

### Role of NK and NKT Cells

NK cells and NKT cells are innate lymphocytes particularly enriched in the liver. Following transplantation, NK cells and NKT cells persist in the liver and blood, unlike donor T cells, B cells, and DCs, which migrate into secondary lymphoid organs and are rejected rapidly. This phenomenon suggests that these cells are resistant to rejection and may contribute to liver tolerance (120). NK cells represent ~30–50% of total lymphocytes in the liver, with constitutive cytolytic functions that are responsible for exogenous pathogen clearance and tumor immunity (121–123). The function of NK cells is controlled by the balance of a series of activatory and inhibitory signals receptors constitutively expressed on the cell surface. NK cells can readily recognize allogeneic cells via a unique self-non-self recognition system, termed “missing self” or “missing ligand” recognition, as MHC-incompatible allogeneic cells lack self MHC class I molecules to engage NK inhibitory receptors (124, 125). However, the exact role of NK cells remains unclear. There is evidence that NK cells contribute to both allograft rejection and tolerance in liver transplantation.

**Figure 2:**
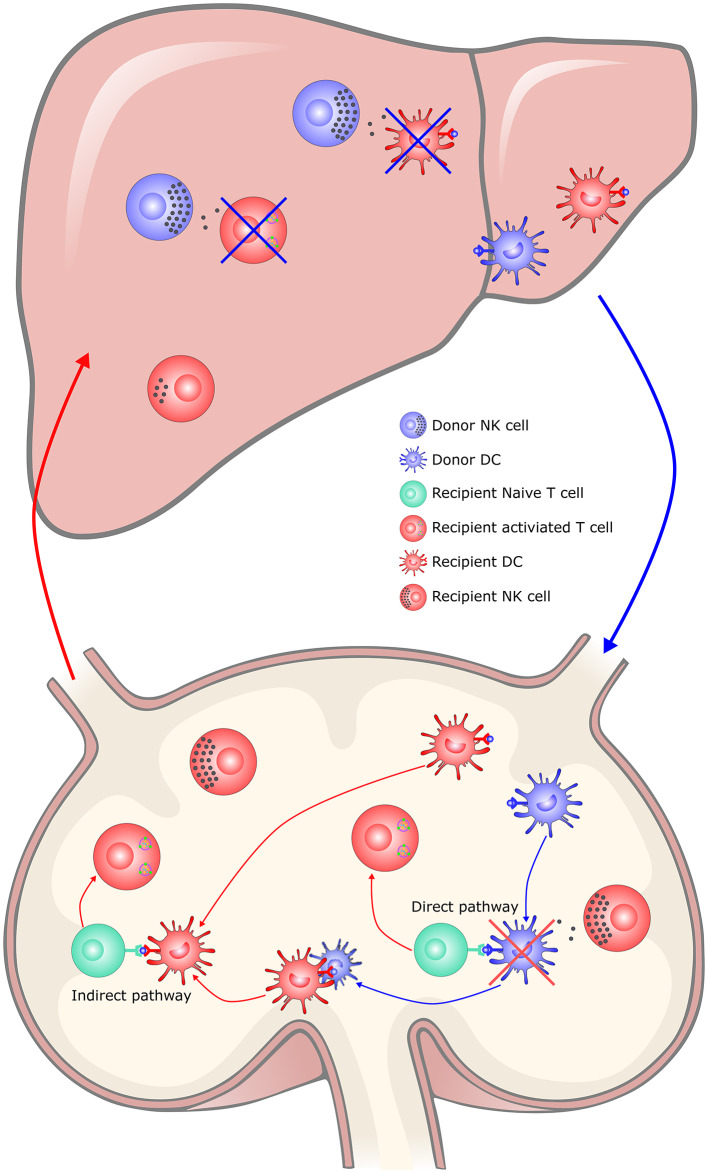
The hypothesis of NK cells in liver transplantation tolerance. In the liver, donor hepatic NK cells promote tolerance, possibly by directly killing recipient immune cells including activated T cells and recipient immature DCs recruited to the allograft, which limited the immune rejection responses. Recipient NK cells would switch to a tolerant phenotype in the tolerogenic hepatic microenvironment. In the secondary lymphoid organs, recipient NK cells kill donor passenger DCs, thereby limiting the activation of T cells by the direct pathway, but favoring the indirect pathway-primed alloreactive T cell response, which contributes to tolerance induction. DC, dendritic cell; NK, natural killer cell; APC, Antigen-presenting cell.

Donor-derived NK cells play a major role in liver tolerance, while recipient-derived NK cells are inclined to reject allografts (126). Following transplantation, donor-derived NK cells migrate from liver grafts into the recipient circulation and sustained for ~2 weeks (127). While some of them may persist within the liver graft for decades (128). Donor hepatic NK cells promote tolerance, possibly by directly killing recipient immune cells including activated T cells, as suggested by an *in vitro* study in which alloantigen-activated T cells express stress-induced NKG2D ligands via the ATM/ATR pathway and became susceptible to autologous NK cell lysis (129). Alternatively, hepatic NK cells may kill recipient immature dendritic cells recruited to the allograft, as suggested by the fact that NK cells lyse immature DCs at sites of inflammation (130). However, there is no clear *in vivo* evidence of the above hypothesis (Figure 2). Infusion of donor liver NK cells could attenuate liver allograft acute rejection and prolong graft survival in rats (131). Although recipient NK cells can mediate rejection by directly lysing allogeneic liver cells, they become phenotypically distinct and functionally less responsive after migrating to the liver, due to the hepatic microenvironment (132). Recently, Jamil et al. reported that recipient NK cells switched to a tolerant phenotype, as reflected by reduced activating receptor expression, cytotoxicity and cytokine production (133). The tolerance of recipient NK cells occurs upstream of the MHC class I-mediated education via perturbation of the IL-12/STAT4 signaling pathway. Outside of the liver, recipient NK cells kill donor passenger DCs, thereby limiting the activation of T cells by the direct pathway, but favoring the indirect pathway-primed alloreactive T cell response, which contributes to tolerance induction (134–137). In addition, clinical data also showed the correlation of NK cells with allograft tolerance in liver transplantation, but information regarding the origin of the NK cells (from the recipient or donor) was lacking (130, 138, 139).

NKT cells are liver-resident lymphocytes that actively patrol the liver. They share features of both NK and T cells and recognize the lipid antigens from either the host or a microbe presented by the non-classical MHC class I-like molecule CD1. NKT cells contribute to most of the immune responses in the liver and play diverse roles in acute liver injury, liver fibrosis and tolerance. NKT cells are believed to promote liver tolerance induction (140). NKT (Jα281) knockout in the donor liver graft was associated with extensive lymphocytic infiltration of portal triads and bile duct epithelium and significantly impaired the graft survival in mouse liver transplantation models (141).

## The Adaptive Immune Tolerance Mechanisms

### The Fate of T Cells

T cells are the major executor of transplantation rejection, doing so by directly destroying allograft cells. The fate of T cells after their activation determines the outcome of transplantation: either allograft tolerance or rejection. Naïve T cells usually lack permission to enter the parenchyma of most organs, due to the lack of the adhesion molecules and chemokine receptors required for adhesion to endothelial cells or subsequent transendothelial migration (142). Naïve T cells circulate in the blood and migrate into secondary lymphoid organs, where they are activated by interacting with DCs. The T cell activation results in adhesion molecule and chemokine receptor upregulation, which allows them to migrate and infiltrate tissues. In the liver, however, the situation is different. The unique liver microenvironment, with its slow blood flow and fenestrated endothelium in the narrow hepatic sinusoids, permits the continuous functional interaction between circulating naive T cells and the diverse hepatic-resident immobile non-professional APCs, as mentioned above. This makes the liver the only non-lymphoid organ that can prime naïve T cells locally independently of DCs and secondary lymphoid organs (143). These non-professional APCs are generally tolerogenic, as reflected in their low expression of MHC molecules, costimulatory molecules and proinflammatory cytokines but rather high expression of coinhibitory molecules and anti-inflammatory cytokines (43, 44, 51, 52, 108, 144–148). They are insufficient to optimally prime the T cells, which leads to the removal of alloreactive T cells, thus promoting tolerance (49, 149–151).

A classic theory refers to the liver as the graveyard of T cells, suggesting the specific ability of the liver to retain and eliminate activated T cells (152, 153). The liver destroys T cells undergoing apoptosis or activated T cells recognizing their antigen *in situ* by clonal deletion, clonal anergy and T cell exhaustion. Activated CD8^+^ T cells perfused through the liver are selectively retained primarily by ICAM-1-expressing hepatocytes, LESCs and KCs and subsequently undergo apoptosis (154). Another important mechanism involved in liver tolerance is the phenomenon that liver-activated T cells may be rapidly destroyed by endosomal/lysosomal-depended degradation following an active invasion of hepatocytes expressing the recognition of their cognate antigens (155). This unique mechanism of peripheral deletion was termed “suicidal emperipolesis” and results in the deletion of at least 75% of antigen-specific CD8^+^ T cells within the first 24 h following activation in the liver.

Other hepatic non-professional APCs, such as LESCs, KCs, and HSCs, also play an important role in liver tolerance through clonal anergy or the deletion of T cells within the hepatic microenvironment. In mouse liver transplantation models, activated CD8^+^ T cells infiltrating the liver allograft were eliminated by locally induced apoptotic cell death (156). Thus, the systemic administration of mouse IL-2, which rescued CD8^+^ T cells from apoptosis, induces acute graft rejection (156, 157). In human liver allografts, prominent T cell apoptosis in the sinusoids was also evident in biopsy specimens (158). Even if some activated CD8^+^ T cells survive these early depletion processes, they may progress to a functionally defective state, known as exhaustion. T cell exhaustion is another pattern of T cell dysfunction that has been frequently studied in the era of chronic viral infection and antitumor immunity (159). T cells become exhausted when encountering a persistent high load of antigens or receiving inhibitory signals, and this condition is characterized by a progressive loss of effector functions and proliferative capacity (160–164). This would most likely happen in the setting of liver transplantation, where the allograft is a large-sized mass and the immunosuppressive microenvironment has an abundant amount of inhibitory signals. Direct evidence of alloreactive CD8^+^ T cell exhaustion was observed following the rapid and extensive activation of T cells early after transplantation in mice (165). However, the contribution of T cell exhaustion to spontaneous liver tolerance needs to be further explored.

CD4^+^ T cells help coordinate immune responses primarily by secreting cytokines that target other immune cells to orchestrate a synchronized immune response (166). After activation, naive CD4^+^ T cells differentiate into distinct T helper cell lineages, including IFN-γ-producing Th1 cells, IL-4-producing Th2 cells, IL-17-producing Th17 cells, and Tregs (167). The cytokine environment dictates the differentiation and conversion of CD4^+^ T cells. The profile of the hepatic microenvironment suppresses the differentiation of proinflammatory Th1 and Th17 cells but favors the skewing of immunosuppressive Th2 and Tregs, which promote allograft tolerance. Tregs are the most well-known suppressor T cells and play an important role in both transplantation tolerance induction and maintenance (168–170). The frequency of Tregs was shown to be increased in liver grafts and host spleens after transplantation (171). The depletion of host Tregs enhanced the T cell response and reduced apoptosis, thereby abrogating spontaneous liver allograft acceptance in a mouse model (171, 172).

## Other Proposed Liver Tolerance Mechanisms

### Role of Soluble Donor MHC-I Molecules

Liver allografts release massive amounts of soluble MHC class I molecules that persist in the recipient circulation at high concentrations (173), which may act as a plausible mechanism of liver transplantation tolerance. The activation of T cells requires the first signal to be provided by the MHC/antigen-peptide complex and the second signal to be provided by the co-stimulatory signal. Stimulation of T cell receptors in the absence of a co-stimulatory signal induces T cells apoptosis (174). Due to the lack of costimulatory molecules, the binding of soluble MHC molecules to T cells leads to tolerance of antigen-specific T cells and is widely used in the study of allogeneic transplantation. A large number of soluble MHC class I molecules are released into the circulatory system in liver transplantation and are involved in inducing immune tolerance and promoting the graft survival (173, 175–178).

Although earlier studies reported that MHC class I-deficient liver allografts were still accepted indefinitely (179), the low immunogenicity due to MHC-deficient makes these studies difficult to interpret. Other studies have shown that soluble MHC molecules inhibit transplant rejection and prolong the graft survival by inhibiting allergic T cells and inducing CTL apoptosis in a dose-dependent manner (180–185). The advent of MHC/antigen-peptide multimer technology has provided T-cell receptor (TCR) with a relatively high-affinity ligand and an effective way of regulating the activation and function of T cells. Soluble MHC class I molecules can also neutralize antibodies by binding to alloantibodies, thereby preventing alloantibody-mediated rejection (175). Furthermore, researchers constructed a mouse soluble MHC dimer and found that it was able to bind to TCR specifically and regulate the TCR expression and phosphorylation, thereby inhibiting the activation and cytotoxicity of T cells (186, 187). Fried et al. reported in 2005 that rat RT1.A-Fc dimers were able to prolong the survival time of heart grafts, suggesting the utility of soluble MHC dimers for inhibiting transplant rejection. pMHC dimer may therefore be useful for inhibiting transplant rejection (188).

### Role of Passenger Leukocytes and Microchimerism

Passenger leukocytes are donor leukocytes that circulate in the recipient's lymphatic tissue after transplantation (189, 190). Microchimerism refers to the persistently low levels of donor cells (<1 per 10^4^ or 10^5^ cells) within the peripheral circulation of the transplant recipient (191). The role of passenger leukocytes and microchimerism in organ transplantation has been controversial. Studies have found that passenger leukocytes are important factors for promoting graft rejection in skin, lung and kidney transplants (192–194). However, in liver transplantation, passenger leukocytes and microchimerism can induce transplant immune tolerance.

Liver passenger leukocytes include B cells, T cells, NK cells, NKT cells, and DCs, which quickly enter the recipient's peripheral circulation and then enter the secondary lymphoid organs after transplantation (120). Previous studies detected a large number of donor passenger leukocytes in recipient secondary lymphoid organs or peripheral blood after liver transplantation in rat, mouse and human models (4, 189, 195). Starzl et al. proposed that liver allografts induced tolerance by the lymphocyte balance between the host and the passenger leukocytes (i.e., the ability to reach a stable chimeric state) (191). Subsequent studies have shown that passenger leukocytes interact with allogeneic CD8^+^ T cells in secondary lymphoid organs, which is an early event in spontaneous liver tolerance (120, 196). Removal of passenger leukocytes by irradiating the donor graft before transplantation results in acute rejection of the graft (196, 197). However, tolerance can be restored by supplementation of liver passenger leukocytes or spleen lymphocytes (196–198). Further research found elevated IL-2 and IFN-γ mRNA levels and apoptotic T cells in transplant-tolerant recipients' secondary lymphoid organs (195, 199). However, other researchers have also suggested that microchimerism is not a major factor in spontaneous liver tolerance, as it fails to predict patients who are suitable for the discontinuation of immunosuppressive therapy (200, 201). Therefore, microchimerism may be the result of tolerance rather than the cause (202). In summary, more research is needed on the role of passenger leukocytes and microchimerism in immune tolerance in liver transplantation.

### Role of the High-Load Antigen Effect

The liver is the largest internal solid organ in the body, which may favor allograft tolerance due to its large tissue mass and high-load alloantigens (MHC molecules). The high-load alloantigens dilutes the finite T cell clones and cytokine levels, leading to a low density of alloreactive T cells and insufficient cytokines, and thus potentially result in exhaustion of T cells and subsequent tolerance. This hypothesis was supported by the results of animal transplantation experiments, which showed that larger skin grafts extended the survival (203, 204), as did multiple organ transplantation (205). In contrast, small grafts have higher rejection rates in rat liver transplant models (206–208). In the reduced-volume liver transplantation model, the recipient's tolerance to the graft increased as did the antigen load, which is consistent with other findings (209). In clinical studies, combined liver-kidney transplantation has been associated with a weaker immune response, lower rejection rate and higher survival rate (9, 10, 210). These findings suggested that a high antigen load may partially account for liver tolerance, although the mechanism remains unclear. Some researchers have proposed plausible explanations for liver tolerance: first, the liver's large size dilutes alloreactive T cells and cytokines, which lower the alloimmune responses (211, 212); second, the liver allograft harbors a large number of passenger leukocytes that may contribute to tolerance as discussed above; last, the high-load antigens favor T cell exhaustion (213).

## Concluding Remarks

**Figure 3:**
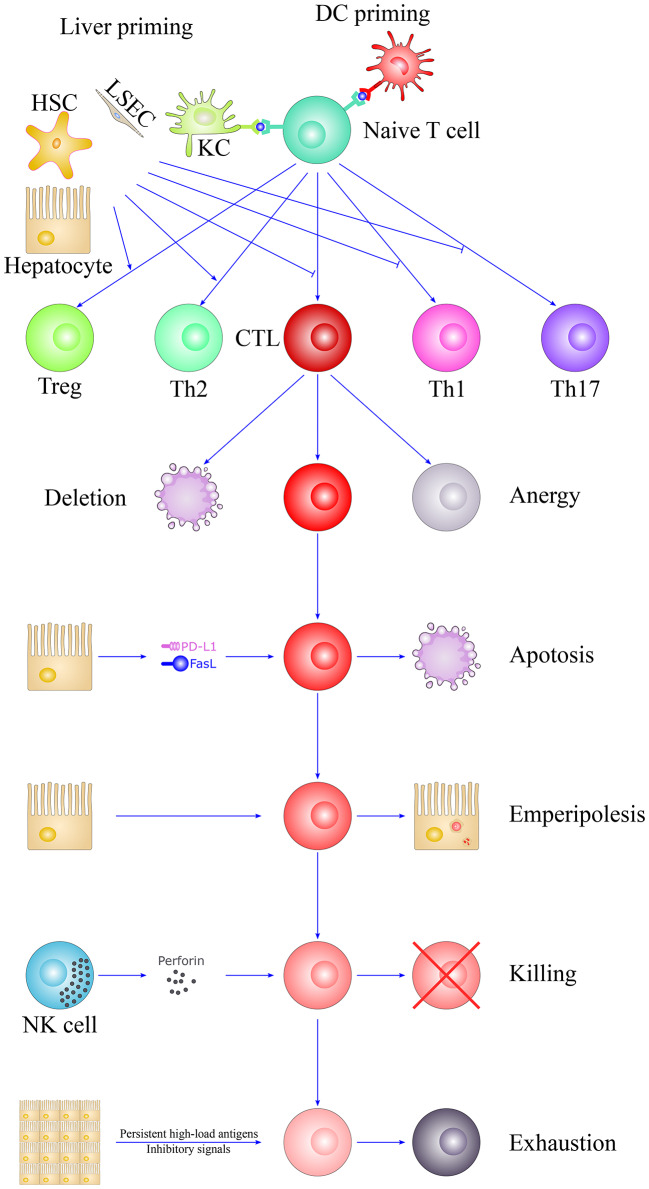
The fate of T cells in liver tolerance. The unique liver microenvironment determines the fate of T cells after activation. T cells were primed by DCs in secondary lymphoid organs or diverse hepatic-resident immobile non-professional APCs in the liver, such as hepatocytes, LSECs, KCs, and HSCs. They are insufficient to optimally prime T cells, which lead to the removal of alloreactive CTLs and suppress the differentiation of proinflammatory Th1 and Th17 cells but favor the skewing of immunosuppressive Th2 and Tregs. The liver is also referred as the graveyard of T cells, suggesting the specific ability of the liver to destroys T cells. Activated T cells would largely eliminate through clonal deletion, clonal anergy, apoptosis, “suicidal emperipolesis,” NK cell killing and T cell exhaustion, thus leading to liver tolerance. HSC, hepatic stellate cell; LSEC, liver sinusoidal endothelial cell; DC, dendritic cell; NK, natural killer cell; CTL, cytotoxic T lymphocyte; KC, Kupffer cell; Th1, T helper cell 1; Th2, T helper cell 2; Th17, T helper cell 17; Treg, Regulatory T cell; PD-L1, programmed death ligand 1; FasL, Fas ligand; APC, Antigen-presenting cell.

The unique tolerogenic hepatic microenvironment is due to the hepatic acinus anatomy and the complex cellular network, thus enabling the local activation of naïve T cells by interacting with diverse hepatic-resident immobile non-professional APCs and resulting in the dysfunction and depletion of T alloreactive T cells. Outside the liver graft, passenger hepatic DCs and recipient NK cells also limit the priming of alloreactive T cells. In addition, soluble donor MHC I molecules, the passenger leukocyte theory and the high-load antigen effect may also be important for achieving liver tolerance. These tolerogenic mechanisms determine the fate of T cells toward clonal deletion, clonal anergy and exhaustion, which eventually leads to tolerance (Figure 3). However, other critical mechanisms may exist, so further studies are yet needed. Understanding the mechanisms underlying the unique immunologically privileged liver organ is valuable for transplantation management and autoimmune disease treatment.

## Author Contributions

All authors listed have made a substantial, direct and intellectual contribution to the work, and approved it for publication.

## Conflict of Interest

The authors declare that the research was conducted in the absence of any commercial or financial relationships that could be construed as a potential conflict of interest.
